# An advanced scheme of compressed sensing of acceleration data for telemonintoring of human gait

**DOI:** 10.1186/s12938-016-0142-9

**Published:** 2016-03-05

**Authors:** Jianning Wu, Haidong Xu

**Affiliations:** School of Mathematics and Computer Science, Fujian Normal University, Fuzhou, 350007 Fujian China

**Keywords:** Compressed sensing, Acceleration data, Gait classification, Gait monitoring and assessment

## Abstract

**Background:**

The compressed sensing (CS) of acceleration data has been drawing increasing attention in gait telemonitoring application. In such application, there still exist some challenging issues including high energy consumption of body-worn device for acceleration data acquisition and the poor reconstruction performance due to nonsparsity of acceleration data. Thus, the novel scheme of compressive sensing of acceleration data is needed urgently for solutions that are found to these issues.

**Methods:**

In our scheme, the sparse binary matrix is firstly designed as an optimal measurement matrix only containing a smallest number of nonzero entries. And then the block sparse Bayesian learning (BSBL) algorithm is introduced to reconstruct acceleration data with high fidelity by exploiting block sparsity. Finally, some commonly used gait classification models such as multilayer perceptron (MLP), support vector machine (SVM) and KStar are applied to further validate the feasibility of our scheme for gait telemonitoring application.

**Results:**

The acceleration data were selected from open Human Activity Dataset of Southern California University (USC-HAD). The optimal sparse binary matrix (a smallest number of nonzero entries is 8) is as strong as the full optimal measurement matrix such as Gaussian random matrix. Moreover, BSBL algorithm significantly outperforms existing conventional CS reconstruction algorithms, and reaches the maximal signal-to-noise ratio value (70 dB). In comparison, MLP is best for gait classification, and it can classify upstairs and downstairs patterns with best accuracy of 95 % and seven gait patterns with maximal accuracy of 92 %, respectively.

**Conclusions:**

These results show that sparse binary matrix and BSBL algorithm are feasibly applied in compressive sensing of acceleration data to achieve the perfect compression and reconstruction performance, which has a great potential for gait telemonitoring application.

## Background

In recent 10 years, the telemonitoring of human gait has been drawing increasing attention in clinical application such as the early identification of at-risk gait of elderly, the assessment of gait during rehabilitation, and so on [1–3]. It is advantageous to easily real-time monitor gait function change of people at home or outdoor environment, and people don’t often need to visit hospital. In most of gait telemonitoring system, the body-worn device equipped with accelerometer is usually served as data acquisition module, and the acquired acceleration data is transmitted, via internet, to remote terminal where further data processing such as gait classification is performed for gait telemonitoring [4–6]. Currently, acceleration data has been widely applied in the field of study on gait monitoring. In gait telemonitoring application, there still exist some challenging issues: energy constrains in body-worn device due to the limited battle life and the poor fidelity of receiving acceleration data at remote terminal [7–9]. Most of pervious studies mainly focused on searching for the technology solutions of energy constraint by designing wireless communication protocol with low energy consumption. Although previous studies can save energy by regulating the data packet size to be sent, they don’t greatly reduce a larger amount of data (i.e. the dominant source of energy waste) during transmission [7, 8]. Recently, some researchers tried to introduce CS method (a novel data compression methodology including compression and reconstruction algorithm) to address existing issues [10–12]. Their basic idea is that in view of gait telemonitoring system, acceleration data processing is divided into two different stages. In the first stage, acceleration data before transmission on body-worn device is compressed at far below Nyquist sampling frequency. In the second stage, reconstruction of acceleration data is performed at remote terminal. For instance, Akimura et al. demonstrated that compressed sensing of acceleration data is feasible in human activity telemonitoring application [10]. In their study, Gaussian measurement matrix and conventional reconstruction algorithm were applied to compress and reconstruct acceleration data, respectively. However, they don’t investigate the effect of nonsparsity of acceleration data on CS reconstruction performance. As we know, acceleration data is characterized by complex dynamic with long-range correlation, and its time series is usually non-stationary and nonlinear [13–15]. Moreover, acceleration data is non-sparse enough in time domain or the transformed domain. So, the nonsparsity of acceleration data must be taken into count in CS reconstruction algorithm that mainly depends on the sparsity of data [16], otherwise reconstruction data with poor fidelity is possibly produced, which largely deteriorates the quality of gait monitoring. Thus, it is need to find the advanced CS reconstruction algorithm for acceleration data with nonsparsity.

Theoretically, acceleration data has the rich data structure such as block structure [17–19], and it is possible to perfectly reconstruct acceleration data by exploiting block sparsity. Recent studies showed that block sparse Bayesian learning (BSBL) algorithm has superior ability to reconstruct non-sparse data by exploiting block sparsity even if data has no distinct block structure [18, 19]. Successful examples include reconstruction of EEG and fetal ECG in telemonitoring setting [20, 21]. To date, no study has been reported that BSBL is used to reconstruct acceleration data. It motivates us to investigate the practicality of application of BSBL for reconstruction of acceleration data with high fidelity.

In CS scheme, the design of measurement matrix is very important to improve CS performance. Currently, the most used measurement matrix mainly includes Gaussian matrix, Bernoulli matrix and sparse binary matrix. In comparison, Gaussian matrix and Bernoulli matrix are theoretically superior to sparse binary matrix. However, they have to be implemented in hardware with high energy consumption [22, 23]. For example, Gaussian random matrix can be generated only when Gaussian random generator is integrated in hardware, thus increasing the hardware complexity and high-computational complexity [22, 23]. In contrast, sparse binary matrix, derived from sub-Gaussian random matrices that is formed by the entries of 0 and 1, has advantages that the efficient implementation of the large matrix multiplication in CS is performed during a short execution time [20, 23]. More importantly, sparse binary matrix can be optimally designed as measurement matrix that only includes a smallest number of nonzero entries. These advantages are greatly contributes to be easily implemented in hardware design for saving more energy. So far, no study has been published that sparse binary matrix is feasibly applied for compressive sensing of acceleration data. Therefore, we try to investigate the feasibility of application of sparse binary matrix in compressive sensing of acceleration data.

So, in this study, we proposed a novel scheme of compressive sensing of acceleration data for gait telemonitoring. That is, sparse binary matrix is designed as an optimal measurement matrix only containing a smallest number of nonzero entries. And BSBL algorithm is introduced to reconstruct nonsparse acceleration data with high fidelity. The acceleration data were selected from an open human activity dataset of University of Southern California (USC-HAD) [24], in order to validate the feasibility of our CS scheme. In addition, three commonly used gait classification models such as support vector machine (SVM) [25], multilayer perceptron (MLP) [26] and KStar [27] are utilized to further test the reconstructed acceleration data with high fidelity for gait monitoring.

The rest of paper is organized as follows. Firstly, we describe the novel CS scheme of acceleration data for gait classification. Secondly, we present the simulation experiment that is made to validate the feasibility of our proposed CS scheme by using the selected USC-HAD data. Thirdly, we further discuss the effectiveness of our CS scheme in gait telemonitoring application according to experimental results. Lastly, the conclusion is presented. 

## The novel CS scheme of acceleration data for gait classification

**Fig. 1:**
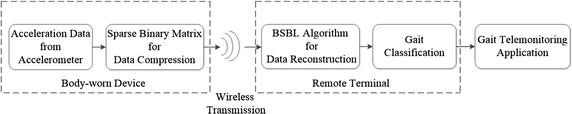
Block diagram of compressive sensing of acceleration data for gait telemonitoring

Compressive sensing theory is proposed by Donoho, and its basic idea is that the best CS performance is achieved by exploring data sparsity. The conventional CS scheme mainly includes sparse representation of data, design of measurement matrix and reconstruction algorithm [11]. In our proposed CS scheme for gait telemonitoring, as shown in Fig. 1, sparse binary matrix is optimally designed as a measurement matrix for acceleration data compression on body-worn device. And BSBL algorithm is introduced to reconstruct acceleration data with high fidelity at remote terminal. This greatly contributes to gait classification with high quality in gait telemonitoring application. The detail of our CS scheme is presented as follows.

### Design of the optimal measurement matrix

According to the CS theory, the raw acceleration data *X* ∊ *R*^*N* × 1^ (*N* denotes the length of data) on body-worn device can be greatly compressed by

1 $$Y = \Phi X$$

where measurement matrix Φ ∊ *R*^*M* × *N*^ (*M* ≤ *N*) and compressed data *Y* ∊ *R*^*M* × 1^ (*M* is data length). Here, sparse binary matrix, as shown in Eq. (2), is selected to be optimally designed as a measurement matrix that contains a smallest amount of nonzero entries, in order to greatly reduce lots of computational resources on body-worn device. For comparison, Gaussian random matrix and Bernoulli random matrix are both selected to further validate the best CS performance from our scheme for acceleration data.

2 $$\Phi = \left( \begin{aligned} 1{ 0 1 0 } \cdots { 1} \hfill \\ 0 { 1 1 0 } \cdots { 0} \hfill \\ \vdots \, \vdots \, \vdots \, \vdots \, \cdots \, \vdots \hfill \\ 1 { 0 0 1 } \cdots { 0} \hfill \\ \end{aligned} \right)$$

### BSBL algorithm for reconstructing acceleration data

In conventional CS reconstruction algorithm, data must satisfy sparse enough in time domain or the transformed domain [11]. That is, data *X* is sparsely represented as *X* = ψα, where ψ ∊ *R*^*N* × *N*^ is sparse basis, and α is the corresponding sparse representation coefficient. And according to Eq. (1), compressed data *Y* = Φ*X* = Φψα. In CS theory, the estimation of sparse coefficient α can be obtained by solving the following *l*_1_ optimization problem

3 $$\hbox{min} \left\| \upalpha \right\|_{1} {\text{subject}}\,{\text{to}}\,Y = \Phi \uppsi \upalpha$$

Therefore, the reconstruction of data $\hat{X}$ can be achieved by the estimated α, i.e.$\hat{X} = \Psi \upalpha \approx X$. The detailed procedure of solution is found in [11].

Unlike the above reconstruction algorithm, BSBL algorithm accurately reconstructs nonsparse data by exploiting block sparsity [17–19]. In this study, acceleration data *X*is considered as a concatenation of a number of blocks, that is, $X = \underbrace {{[x_{1} , \ldots ,x_{{h_{1} }} }}_{{x_{1}^{T} }}, \ldots ,\underbrace {{x_{{h_{l - 1} + 1}} , \ldots ,x_{{h_{l} }} ]^{T} }}_{{x_{l}^{T} }}$ where *l* is a number of the randomly partitioned blocks, and *h*_*i*_ denotes the partitioned block size. In BSBL algorithm for reconstructing acceleration data, each partition block *X*_*j*_ ∊ *R*^*l* × 1^ satisfies the parameterized multivariate Gaussian distribution:

4 $$p\left( {X_{j} ;\uplambda_{j} ,b_{j} } \right) \sim N\left( {0,\uplambda_{j} b_{j} } \right),\quad j = 1,2, \ldots ,l$$

where the unknown positive parameters λ_*j*_ is used to capture block sparsity, and λ_*j*_ = 0 means that the *j*-*th* block is zero. The unknown parameter $b_{j} \in R^{{d_{j} \times d_{j} }}$ is a positive definite matrix, and it describes the correlation among elements within the *j*-th block. Here, all partitioned block are assumed to satisfy mutual uncorrelated, and the prior density of *X* is defined as

5 $$p\left( {X;\left\{ {\uplambda_{j} ,b_{j} } \right\}} \right) \sim N\left( {0,\sum_{x} } \right) \quad where\,\sum\nolimits_{x} { = \left\{ \begin{aligned} \uplambda_{1} b_{1} ,0, \ldots \ldots ,0 \hfill \\ 0,\uplambda_{2} b_{2} ,0, \ldots ,0 \hfill \\ \ldots \ldots \ldots \ldots \ldots \ldots \hfill \\ 0,0, \ldots \ldots \ldots ,\uplambda_{l} b_{l} \hfill \\ \end{aligned} \right\}}$$

In view of acceleration data contaminated by noise, compressed data *Y* ∊ *R*^*M* × 1^ is defined as

6 $$Y = \Phi X + Z$$

where *Z* ∊ *R*^*M* × 1^ denotes noise, and it satisfies Gaussian distribution of N(0, ρ*I*)where ρ is a positive scalar and *I* ∊ *R*^*M* × 1^is an identity matrix. Based on *Y* ∊ *R*^*M* × 1^, the posterior density of *X* is defined as Gaussian distribution

7 $$p\left( {{X \mathord{\left/ {\vphantom {X {Y;\uprho ,\left\{ {\uplambda_{j} ,b_{j} } \right\}_{j = 1}^{l} }}} \right. \kern-0pt} {Y;\uprho ,\left\{ {\uplambda_{j} ,b_{j} } \right\}_{j = 1}^{l} }}} \right) = {\rm N}\left( {\upmu_{\Uptheta } ,\sum\nolimits_{\Uptheta } {} } \right)$$

where the mean value $\upmu_{\Uptheta } = \sum\nolimits_{x} {\Phi^{T} \left( {\uprho I + \Phi \sum\nolimits_{x} {\Phi^{T} } } \right)^{ - 1} Y}$, and the value of covariance matrix $\sum\nolimits_{\Uptheta } = \left( {\sum\nolimits_{x}^{ - 1} {} + \frac{1}{\uprho }\Phi^{T} \Phi } \right)^{ - 1}$.

So, reconstruction of acceleration data $\hat{X}$ is achieved when all parameters (ρ, λ_*j*_, *b*_*j*_) are available, i.e. $\hat{X} \approx \upmu_{\Uptheta }$. In BSBL framework, all parameters (ρ, λ_*j*_, *b*_*j*_) are obtained by optimal learning algorithms [18, 19]. In this study, we select the bound-optimization BSBL algorithm (i.e. BSBL-BO) due to its faster convergence speed. The detail procedure for solution is presented in Appendix 1.

For comparison, some conventional CS reconstruction algorithms were selected to validate superior ability of BSBL algorithm for reconstructing acceleration data. These selected algorithms include: (1) algorithms without block structure such as orthogonal matching pursuit (OMP) [28], basis pursuit (BP) [29], subspace pursuit (SP) [30], smoothed *l*_0_-norm (SL0) [31]; (2) algorithms with block structure such as dynamic group sparsity (DGS) [32], structured orthogonal matching pursuit (SOMP) [33], Group Lasso [34], and these algorithms only consider the prior knowledge of block partition.

### Evaluation criteria for the proposed CS scheme

In this study, the assessment of performance of our scheme is implemented according to the following common criteria [18, 20, 22, 23]:

1. Compression ratio (CR): it is used to quantitatively evaluate the ability of compression of acceleration data, and is defined as
  8 $$CR = \frac{N}{M} \times 100 \, \%$$
2. Normalized mean square error (NMSE): it is employed to assess the performance of reconstruction of acceleration data, and is defined as
  9 $$NMSE(X,\hat{X}) = \frac{{\left\| {X - \hat{X}} \right\|_{2}^{2} }}{{\left\| X \right\|_{2}^{2} }}$$
3. Signal-to-noise ratio (SNR): it is only used to measure the ability of the reconstruction of acceleration data, and is defined as
  10 $$SNR = 10\log_{10} \frac{{\sum\nolimits_{n = 1}^{N} {X^{2} (n)} }}{{\sum\nolimits_{n = 1}^{N} {(X(n) - \hat{X}(n))^{2} } }}$$
4. Pearson correlation coefficient: it is also used to evaluate the reconstruction performance by testing the similarity difference between the raw data and the reconstruction data, and is defined as
  11 $$R = \frac{{\sum\nolimits_{i = 1}^{N} {\left( {X_{i} - \bar{X}} \right)\left( {\hat{X}_{i} - \bar{\hat{X}}} \right)} }}{{\sqrt {\sum\nolimits_{i = 1}^{N} {\left( {X_{i} - \bar{X}} \right)^{2} } } \sqrt {\sum\nolimits_{i = 1}^{N} {\left( {\hat{X}_{i} - \bar{\hat{X}}} \right)^{2} } } }} \quad i = 1,2, \ldots ,N$$

where $\bar{\hat{X}}$ denotes the mean value.

### Gait classification models based on the reconstructed acceleration data

Three commonly used gait classification models such as SVM, MLP, and KStar were selected to further test the reconstructed acceleration data with high fidelity for gait monitoring. SVM classification model is derived from the Vapnik–Chervonenkis theory and structural risk minimization [25]. Its basic idea is that the data to be classified can be firstly mapped into the high-dimensional feature space via kernel function, and then an optimal separating hyperplane is constructed between the classes in the mapped space. The multilayer perceptron is also an effective classification model based on artificial neural networks, and it usually uses the back propagation algorithm to construct classification model [26]. KStar is a common classification model based on k-nearest neighbors framework, and its basic idea is to measure the difference between input sample by using entropy-based distance [27]. A detailed description of these classification algorithms are found in [25–27], respectively.

In order to improve classification performance, some good gait features containing more separate information are selected from the reconstructed acceleration data according to some common statistical parameters [5, 9, 10, 13, 14]: Standard deviation (SD), Skewness, Kurtosis and Pearson correlation coefficient. These selected parameters are defined as follows:

12 $$SD = \sqrt {\frac{1}{N}\sum\limits_{i = 1}^{N} {\left( {\hat{X}_{i} - \bar{\hat{X}}_{i} } \right)}^{2} }$$

13 $$Skewness = \frac{{N\sum\nolimits_{i = 1}^{N} {\left( {\hat{X}_{i} - \bar{\hat{X}}_{i} } \right)^{3} } }}{{\left( {N - 1} \right)\left( {N - 2} \right)SD^{3} }}$$

14 $$Kurtosis = \left( {\frac{{N\left( {N + 1} \right)}}{{\left( {N - 1} \right)\left( {N - 2} \right)\left( {N - 3} \right)}}\frac{{\sum\nolimits_{i = 1}^{N} {\left( {\hat{X}_{i} - \bar{\hat{X}}_{i} } \right)^{4} } }}{{SD^{4} }}} \right) - \frac{{3\left( {N - 1} \right)^{2} }}{{\left( {N - 2} \right)\left( {N - 3} \right)}}$$

In addition, Pearson correlation coefficient is defined in Eq. (11).

Also, the prediction ability of gait classification model is evaluated according to three statistical measures [16, 35]: Accuracy (Acc), Sensitivity (Sen), and Specificity (Spe). The above measures are defined as follows:

15 $$Accuracy = \frac{TP + TN}{TP + FP + TN + FN} \times 100 \, \%$$

16 $$Sensitivity = \frac{TP}{TP + FN} \times 100 \, \%$$

17 $$Specificity = \frac{TN}{TN + FP} \times 100 \, \%$$

where TP refers to the number of true positive; FP is the number of false positive; TN denotes the number of true negative; FN is the number of false negative.

## Experimental results

### Acceleration data dataset

In this study, the acceleration data from an open USC-HAD (http://www-scf.usc.edu/~mizhang/datasets.htm) [24] were selected to validate the feasibility of our proposed CS scheme. The USC-HAD includes acceleration data of 14 subjects with 12 low-level activities such as walk forward, walk left, walk right, go upstairs, go downstairs, run forward, jump up and down, sit and fidget, stand, sleep, elevator up, and elevator down. Each activity was asked to perform on different days at various indoor and outdoor locations for capturing more information related to day-to-day activity variations. The acceleration data was acquired by a MotionNode (e.g. a high-performance factory-calibrated inertial sensing device) which integrates a 3-axis accelerometer (±6 g) and a 3-axis gyroscope (±500 dps). MotionNode was asked to wear on the front right hip of each subject and the sample ratio was set to 100 Hz. For each activity, each participant was asked to perform 5 trials.

### Evaluation of sparse binary matrix for CS of acceleration data

**Fig. 2:**
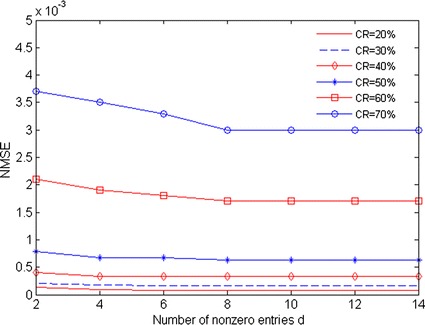
Results of NMSE versus d for different CRs

Firstly, we evaluate the effect of sparse binary measurement matrix containing the smallest number of nonzero entries on CS performance. In the experiment, sparse binary matrix only includes *d* nonzero entries in each column, and the smallest number of nonzero entries*d*is determined according to the best CS performance while compression ratios are varied. The acceleration data length is set to 512 points (i.e. *N* = 512). BSBL-BO and NMSE are used as reconstruction algorithm and evaluation criterion, respectively. The results are shown in Fig. 2, and it is obviously that while compression ratios are varied, NMSE varies with the value of *d*. When compression ratio is less than 30 %, NMSE slightly changes during the increase of the value of *d*. In contrast, when compression ratio reaches 50 %, NMSE increases with the value of *d*. Especially, when the number of *d* exceeds 8, NMSE still keeps lower and stable. These results illustrate that sparse binary matrix containing a small number of nonzero entries is feasible for compressive sensing of acceleration data. In addition, when compression ratio is more than 80 %, NMSE significantly grows, suggesting that higher compression ratios possibly destroy CS performance.

**Fig. 3:**
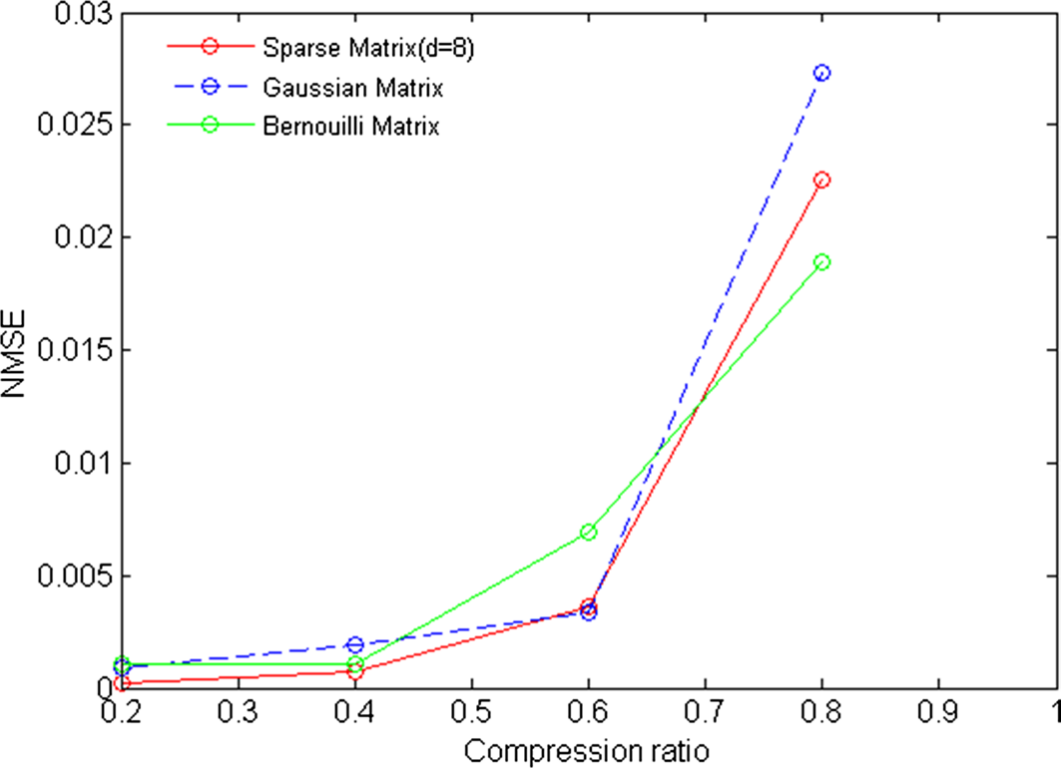
Results of NMSE versus CRs for three different measurement matrices

For comparison, Gaussian random matrix and Bernoulli random matrix are performed in the experiment. Both selected matrices satisfy independent identically distributed, and the entries of each matrix are obtained by sampling its corresponding distribution. The comparative results are shown in Fig. 3. It is apparent that NMSE from three matrices all increase with compression ratios. In comparison, NMSE from our designed sparse binary matrix is lower when compression ratio is less than 60 %. Especially, while compression ratio is close to 40 %, NMSE from three matrices is almost same. These results show that our designed sparse binary matrix is as strong as the full random matrices in compressive sensing of acceleration data.

### Evaluation of the performance of BSBL reconstruction algorithm

**Fig. 4:**
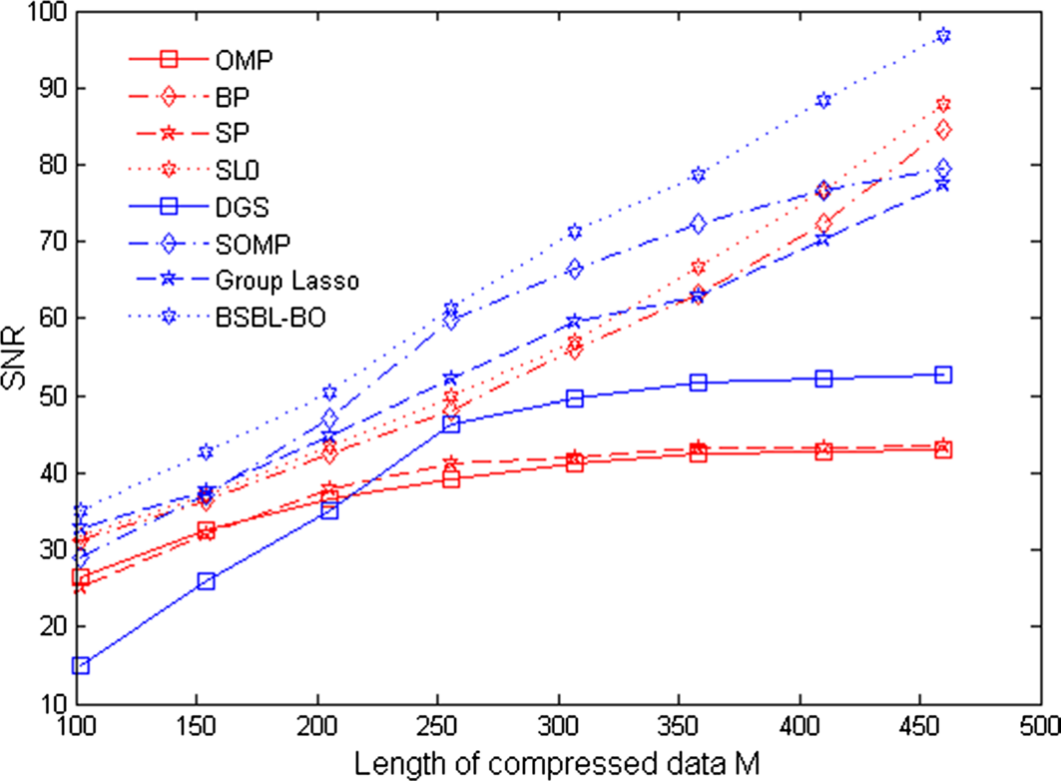
Results of SNR versus M

**Fig. 5:**
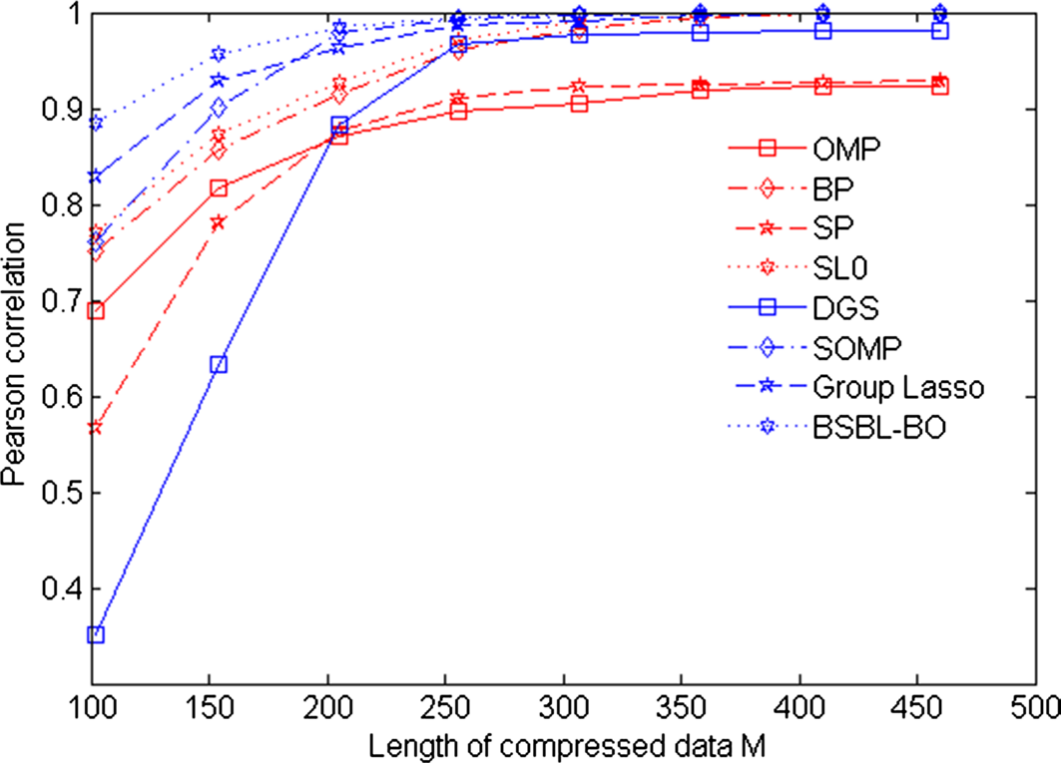
Results of Pearson correlation coefficient versus M

Next, we evaluate the reconstruction performance of BSBL algorithm. In this experiment, acceleration data length is fixed to 500 (i.e. *N* = 500), and it is divided into 25 blocks, each block containing 20 points. Our designed sparse binary matrix and discrete cosine group dictionary are used as measurement matrix and sparse basis ψ, respectively. For comparison, some conventional CS reconstruction algorithms such as OMP, BP, SP, SL0, DGS, SOMP, and Group Lasso are performed. Pearson correlation coefficient and signal-to-noise ratio (SNR) are selected as evaluation criteria, respectively. Each reconstruction algorithm is repeated for 20 times. The comparative results are shown in Figs. 4 and 5, respectively. As shown in Fig. 4, SNR from most of algorithms increases with the value of*M*, except that SNR from OMP, BP and DGS decrease. In comparison, SNR from BSBL-BO algorithm is maximal. Similarly, as shown in Fig. 5, it is obviously that Pearson correlation coefficient values from all reconstruction algorithms increase with the value of*M*. In comparison, Pearson correlation coefficient from BSBL-BO is higher when the value of *M* is less than 350. Especially, from Fig. 4 and 5, it is apparent that when a number of *M*is close to 250, the reconstruction performance of BSBL-BO algorithm is best. In addition, Table 1 also presents that the comparative results among all reconstruction algorithms when compression ratio is set to 50 %. As shown in Table 1, BSBL-BO algorithm reaches the maximal SNR value of 70 dB while its execution time is higher. This is because BSBL algorithm needs a large number of iterations for finding fewer sparse blocks hidden in acceleration data. In conclusion, these results illustrate that BSBL algorithm feasibly reconstruct acceleration data with high fidelity by exploiting block sparsity.

**Table 1:** Performance comparison between different reconstruction algorithms

Reconstruction algorithms	SNR (dB)	Reconstruction time (s)	CR (%)
OMP	37	0.0078	50
BP	48	0.6363	50
SP	37	0.0079	50
SL0	52	0.2465	50
DGS	51	0.0780	50
Group Lasso	55	0.0875	50
SOMP	59	0.0796	50
BSBL-BO	70	0.6412	50

### Evaluation of the effect of our proposed CS scheme on gait classification

With the reconstructed data, different gait classification models based on SVM, MLP and KStar were implemented to evaluate the effectiveness of our CS scheme in gait telemonitoring application. In the experiment, the acceleration data from all subjects in USC-HAD dataset were selected. The window was set to be length *N* = 512 with 50 % overlap [16], and 10 windows were randomly extracted from each subject’ data. That is, for each pattern, sample data size of each subjects are 10, and each sample data length is fixed to 512 (i.e. *N* = 512). Based on four statistical parameters from each axis data, a gait pattern is defined as 12 dimensions feature vector. In this experiment, MLP with momentum back propagation algorithm is used, and SVM containing sequential minimal optimization algorithm with the polynomial kernel function is employed. All classification algorithm programs are developed in Matlab 7.0 using WEKA [36], and they are performed on computer with Intel(R) Core(TM) i5-3470 3.20 GHz CPU, 4.00 GB RAM and Windows 7 operating system.

Firstly, we select two gait patterns such as up-stairs and down-stairs for testing classification performance. To effectively evaluate the generalization ability across subjects, 10 subjects’ data patterns including 100 upstairs and 100 downstairs were randomly selected as training set. In the process of training classification model, a tenfold cross validation scheme was implemented due to the limited data size. That is, the selected 200 gait patterns data were randomly divided into 10 subsets, each subset including 10 upstairs data and 10 downstairs data. 9 subsets were arbitrarily selected as training set while the remaining one was used to test. The above step is repeated until each subset was employed to test. After classification model was obtained, the remaining 4 subjects’ data including 40 upstairs and 40 downstairs were used to test its generalization ability. It is noted that the remaining 4 subjects’ data are not included in training set.

Table 2 presents the best results from three selected classification models. As shown in Table 2, it is apparent that all generalization performance from three selected models are higher while BSBL algorithm is performed. In comparison, classification performance of MLP is best, and it reaches same maximum value of accuracy (95 %), sensitivity (95 %), and specificity (95 %), suggesting that MLP with momentum back propagation algorithm can capture more additional discriminatory information regarding gait patterns. These results illustrate that BSBL algorithm has superior ability to reconstruct acceleration data with high fidelity for gait classification with high quality.

**Table 2:** The best prediction results from classification models with all reconstruction algorithms

Reconstruction algorithms	Classification models	Prediction results				
		ACC (%)	Upstairs		Downstairs	
			Sen (%)	Spe (%)	Sen (%)	Spe (%)
OMP	KStar	62.5	60.9	64.7	64.7	60.9
	SVM	65	60	66.7	66.7	60
	MLP	67.5	65.2	70.6	70.6	65.2
BP	KStar	67.5	65.2	70.6	70.6	65.2
	SVM	70	65.4	78.6	78.6	65.4
	MLP	72.5	73.7	71.4	71.4	74.7
SP	KStar	55	87.5	57.1	57.1	87.5
	SVM	62.5	60.9	64.7	64.7	60.9
	MLP	65	62.5	68.8	68.8	62.5
SL0	KStar	70	65.4	78.6	78.6	65.4
	SVM	72.5	69.6	76.5	76.5	69.6
	MLP	75	67.9	91.7	91.7	67.9
DGS	KStar	67.5	66.7	68.4	68.4	66.7
	SVM	72.5	69.6	76.5	76.5	69.6
	MLP	75	75	75	75	75
Group Lasso	KStar	72.5	69.6	76.5	76.5	69.6
	SVM	75	72.7	77.9	77.9	72.7
	MLP	77.5	72	86.7	86.7	72
SOMP	KStar	77.5	76.5	79	79	76.2
	SVM	85	79.2	93.8	93.8	79.2
	MLP	87.5	85.7	89.5	89.5	85.7
BSBL-BO	KStar	80	82.6	94.1	94.1	82.6
	SVM	92.5	90.5	94.7	94.7	90.5
	MLP	95	95	95	95	95

In addition, the selection of optimal parameters is important for best classification performance. Here, the optimal parameters are determined according to the best performance because the optimal value of each parameter varies with different values of other parameters in each classification model. The optimal parameters selected in each model are presented as follows. In MLP, the number of learning epochs is 600. The learning rate is 0.3, and the momentum factor is 0.5. In SVM, the regularization parameter is 1 and the degree of polynomial is 2. In KStar, the parameter for global blending is 20. A detail description of these parameters can be found in [36].

Next, we employ the above optimal MLP model to classify seven different gait patterns such as walk forward, walk left, walk right, go upstairs, go downstairs, run forward, sitting, in order to further evaluate the practicability of our scheme for gait monitoring. In this experiment, to effectively evaluate the generalization ability across subjects, the gait pattern data from 13 subjects are randomly selected as training set containing all sample size of 910 (i.e. 130 walk forward data, 130 walk left data, 130 walk right data, 130 upstairs, 130 downstairs, 130 run data and 130 sitting data). In the process of training model, a tenfold cross validation scheme was implemented, that is, the selected data is randomly divided into 10 subsets, each subset containing 13 walk forward data, 13 walk left data, 13 walk right data, 13 upstairs data, 13 downstairs data, 13 run data and 13 sitting data. 9 subsets were randomly selected to train while the remaining one was used to test. The above step was repeated until each subset was used to test. After the classification model was obtained, the remaining one subject data including all sample size of 70 (i.e. 10 walk forward data, 10 walk left data, 10 walk right data, 10 upstairs, 10 downstairs, 10 run data and 10 sitting data) was used to test the generalization ability. Here, each subject data has to be used to test generalization ability. Finally, the whole averaged classification result is obtained for all subjects. Table 3 gives the confusion table for classification of seven gait patterns. Here, it is noted that the entry in the *i*th row and *j*th column refers to the count of gait pattern belonging to class *i* that is classified as class *j*. As shown in Table 3, the whole averaged classification accuracy across all gait patterns reaches 92 %, suggesting that gait classification performance is superior. In term of individual patterns, sitting pattern can gain the maximal precision and recall value of 99 %, and sitting pattern is classified accurately. For upstairs pattern, it is noticed that its precision is near to 91 % whereas its recall is only 83 %. The possible reason is that some samples of upstairs are easily misclassified as walk forward, but some samples of walk forward is not misclassified as upstairs. In conclusion, these results show that our proposed CS scheme can provide more valuable information regarding gait pattern change for gait classification with higher quality.

**Table 3:** The confusion table for classification of seven different gait patterns

	Walk forward	Walk left	Walk right	Go up-stairs	Go down-stairs	Run	Sitting	Total	Recall (%)
Walk forward	125	4	5	5	1	0	0	140	89
Walk left	6	125	2	2	5	0	0	140	89
Walk right	2	3	134	0	1	0	0	140	96
Go up-stairs	10	6	3	116	3	1	1	140	83
Go up-stairs	1	3	4	4	127	1	0	140	91
Run	0	1	0	0	3	136	0	140	97
Sitting	1	0	0	1	0	0	138	140	99
Total	145	142	148	128	140	138	139		
Recall (%)	86	88	91	91	91	98	98		

## Discussions

Currently, the novel scheme of compressive sensing of acceleration data is needed urgently for addressing some challenging issues such as energy constraint and poor reconstruction performance in gait telemonitoring application. In the present study, in view of energy constraint on body-worn device, we investigate the feasibility of sparse binary matrix in compressive sensing of acceleration data. As shown in Figs. 2 and 3, our designed sparse binary matrix containing few nonzero entries can feasibly produce the almost same best CS performance as some common used full optimal random matrices such as Gaussian matrix. This greatly contributes to saving more computational resources for hardware design of body-worn device. Besides, we also find that higher compression ratio possibly destroy the proposed CS performance. The possible reason is that higher compression ratio worsen the maximal incoherent measurement between sparse binary measurement matrix Φ and the sparse basis ψ [11].

In addition, in view of acceleration data with nonsparsity, we also investigate the practicability of BSBL algorithm for reconstructing acceleration data with high fidelity by exploiting block sparsity. As seen from Figs. 4, 5 and Table 1, BSBL-BO algorithm significantly outperform existing CS reconstruction algorithms such as OMP, BP, SP, SL0, DGS, SOMP and Group Lasso. This is because BSBL algorithm has superior ability to capture few unknown nonzero blocks by exploiting intra-block correlation in acceleration data. In contrast, existing CS reconstruction algorithms only rely on the priori knowledge of data block partition, not to provide valuable insight into specific types of possible intra-block information [18, 37, 38]. Similar results of study on EEG telemonitoring have been found in [20].

In this study, with the reconstructed acceleration data, we further evaluate the effectiveness of our proposed CS scheme in gait telemonitoring application by using gait classification. As shown in Tables 2,3, all selected gait classification models can produce superior generalization performance. This is because BSBL algorithm takes advantage of exploring intra-block correlation in acceleration data to gain more valuable information associated with the spatial, temporal and dynamic correlations of human gait, which greatly contribute to improving gait classification performance. In addition, this study shows that MLP is best in all gait classification models. The possible reason is that MLP with back propagation algorithm can significantly improve the learning speed of optimization algorithm for acceleration data, thus avoiding the local optimal solution of classification algorithm. Similar finding has been reported in [35].

## Conclusion

In this study, we propose a novel scheme of compressive sensing of acceleration data for gait telemonitoring. In such scheme, sparse binary matrix is feasibly designed as measurement matrix only containing few nonzero entries, which greatly contribute to saving lots of computational resources on body-worn device. Also, BSBL algorithm is feasibly applied to reconstruct acceleration data with high fidelity by exploiting block sparsity, which is very helpful for gait classification with high quality. This study has a great potential for gait telemonitoring application. More effort should be exerted to find the effective method that can significantly improve the ability of compressive sensing of acceleration data while compressed ratio is higher.

## Appendix

In BSBL framework, all unknown parameters (ρ, λ_j_, *b*_j_) is estimated by maximum likelihood procedure that minimizes the following cost function

$$L\left( {\uprho ,\left\{ {\uplambda_{j} ,b_{j} } \right\}} \right) = \log \left| {\uprho I + \Phi \sum\nolimits_{x} {} \Phi^{T} } \right| + Y^{T} \left( {\uprho I + \Phi \sum\nolimits_{x} {} \Phi^{T} } \right)^{ - 1} Y$$

In BSBL-BO algorithm, the learning rules for parameters (ρ, λ_j_, *b*_j_) is defined as follows:

$$\uprho \leftarrow \frac{{\left\| {Y - \Phi \upmu_{\Uptheta } } \right\|_{2}^{2} + Tr\left( {\sum\nolimits_{\Uptheta } {\Phi^{T} \Phi } } \right)}}{M}$$

$$\uplambda_{j} \leftarrow \sqrt {\frac{{X_{j}^{T} b_{j}^{ - 1} X_{j} }}{{Tr((\Phi^{j} )^{T} (\varSigma_{Y}^{*} )^{ - 1} \Phi^{j} b^{j} )}}} \quad \text{where} \quad \sum\nolimits_{Y}^{*} { = \uplambda I + \Phi \sum\nolimits_{x}^{*} {\Phi^{T} } }$$

$$b_{j} \leftarrow \frac{1}{l}\sum\limits_{i = 1}^{l} {\frac{{\varSigma_{\Uptheta }^{j} + u_{\Uptheta }^{j} (u_{\Uptheta }^{j} )^{T} }}{{\uplambda_{j} }}}$$

And then, the optimal parameters (ρ, λ_j_, b_j_) are solved according to the defined learning rules. So, reconstruction of acceleration data $\hat{X}$ is obtained by calculating

$$\hat{X} = \upmu_{\Uptheta } = \sum\nolimits_{x} {} \Phi^{T} \left( {\uprho I + \Phi \sum\nolimits_{x} {} \Phi^{T} } \right)^{ - 1} Y$$
